# The poor reliability of thermal conductivity data in the aerogel literature: a call to action!

**DOI:** 10.1007/s10971-023-06282-9

**Published:** 2024-01-03

**Authors:** Wim J. Malfait, Hans-Peter Ebert, Samuel Brunner, Jannis Wernery, Sandra Galmarini, Shanyu Zhao, Gudrun Reichenauer

**Affiliations:** 1 Laboratory for Building Energy Materials and Components, Empa - Swiss Federal Laboratories for Materials Science and Technology, Dübendorf, Switzerland; 2 Center for Applied Energy Research (CAE), Würzburg, Germany

**Keywords:** Data integrity, Superinsulation, Sample conditioning, Quality control

## Abstract

Aerogels are an exciting class of materials with record-breaking properties including, in some cases, ultra-low thermal conductivities. The last decade has seen a veritable explosion in aerogel research and industry R&D, leading to the synthesis of aerogels from a variety of materials for a rapidly expanding range of applications. However, both from the research side, and certainly from a market perspective, thermal insulation remains the dominant application. Unfortunately, continued progress in this area suffers from the proliferation of incorrect thermal conductivity data, with values that often are far outside of what is possible within the physical limitations. This loss of credibility in reported thermal conductivity data poses difficulties in comparing the thermal performance of different types of aerogels and other thermal superinsulators, may set back further scientific progress, and hinder technology transfer to industry and society. Here, we have compiled 519 thermal conductivity results from 87 research papers, encompassing silica, other inorganic, biopolymer and synthetic polymer aerogels, to highlight the extent of the problem. Thermal conductivity data outside of what is physically possible are common, even in high profile journals and from the world’s best universities and institutes. Both steady-state and transient methods can provide accurate thermal conductivity data with proper instrumentation, suitable sample materials and experienced users, but nearly all implausible data derive from transient methods, and hot disk measurements in particular, indicating that under unfavorable circumstances, and in the context of aerogel research, transient methods are more prone to return unreliable data. Guidelines on how to acquire reliable thermal conductivity data are provided. This paper is a call to authors, reviewers, editors and readers to exercise caution and skepticism when they report, publish or interpret thermal conductivity data.

**Graphical Abstract:**

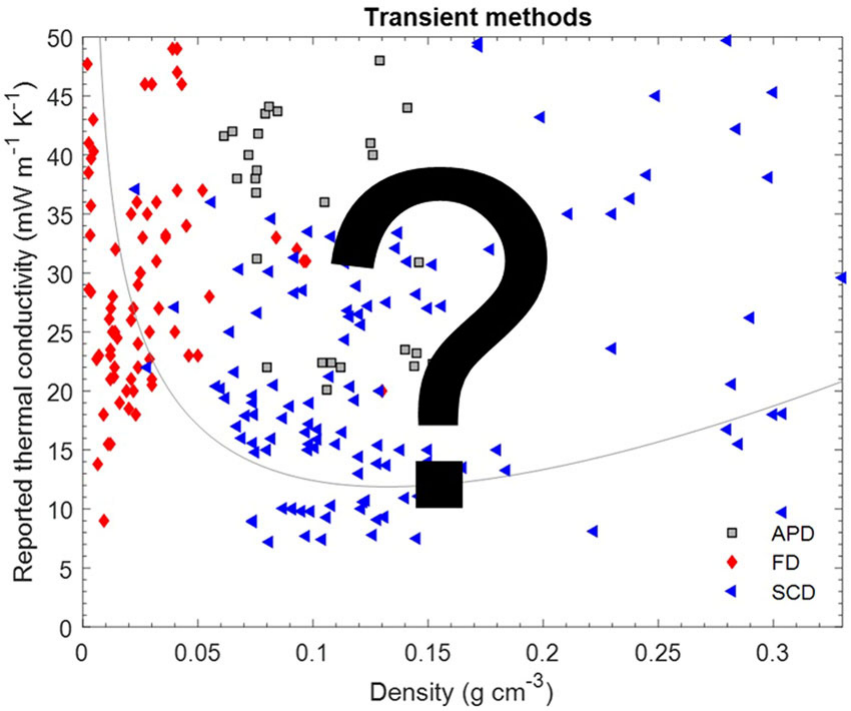

## Highlights

- Aerogels’ ultra-low thermal conductivity for thermal superinsulation applications.
- Over 500 aerogel thermal conductivity data were compiled.
- Aerogel literature contains many unrealistic thermal conductivity data.
- Steady-state methods are more reliable data than transient methods.

## Introduction

Aerogels represent an exceptional class of porous materials [1–3] with potential applications for thermal insulation [4–6], drug delivery [7, 8], tissue engineering [9], catalysis [10], acoustic insulation [11, 12], solar-powered water generation [13] and environmental remediation [14]. These remarkable materials can be produced from diverse substances, including silica, other metal oxides, metals, carbons, synthetic polymers and biopolymers. However, among all of these materials and applications, silica aerogels for thermal insulation are the only material and application that already made strong inroads in the real-world, with rapidly growing markets in industrial and pipeline insulation, building insulation and thermal separators in battery-powered electric vehicles [15, 16]. The insulation application valorizes the record-breaking low thermal conductivity of certain types of silica aerogels, which enables the same insulation performance for less than half the thickness compared to conventional insulation materials such as mineral wool [17]. Thermal insulation is the most prominent aerogel application in the scientific literature, with many papers explicitly targeting it in their titles and abstracts.

Despite nearly a century of aerogel research [18], the definition of what qualifies as an aerogel and what does not remains a topic of debate. The existing definition by IUPAC is not helpful as it limits aerogels to microporous solids such as zeolites and microporous silica, i.e. far removed from how the term is generally understood and used. Historical definitions have been based on drying technique: aerogel from supercritical drying (SCD), xerogels from evaporative ambient pressure drying (APD) and cryogels from freeze-drying (FD). Recently, property-based definitions have been proposed [19], often stressing the mesoporous nature of aerogels. However, in practice, the scientific literature contains numerous papers referring to macroporous materials produced by freeze-drying as aerogels. This lack of a clear and universally accepted definition, coupled with the broad range of materials labeled as aerogels, leads to confusion. Editors, reviewers and readers often assume that any aerogel can exhibit the ultra-low thermal conductivity associated with certain types of thermally optimized, mesoporous aerogels, and as a result, they do not exercise sufficient skepticism when evaluating the validity of thermal conductivities claimed in the literature.

## Heat transfer mechanisms in porous materials

Heat transfer in porous materials occurs via the solid backbone, the gas phase and by radiative transfer. In the simplest case, all heat transfer mechanisms are treated independent of each other and can be described using a diffusion model, which defines specific thermal conductivities for each mechanism according to Fourier’s law. Assuming that no coupling effects between heat transfer via the gaseous and solid phases is present [20], and neglecting convection, the local heat flux density (*q)* for a local temperature gradient (∇T) is determined by summing the individual conductivity values and:

1 $$q = - \lambda _{{{{\mathrm{tot,eff}}}}}\left( {T,\,p_g} \right) \cdot \nabla {{{\mathrm{T}}}}$$

with the total effective thermal conductivity:

2 $$\lambda _{{{{\mathrm{tot,eff}}}}}\left( {T,\,p_g} \right) = \lambda _s\left( {{{\mathrm{T}}}} \right) + \lambda _g\left( {T,\,p_g} \right) + \lambda _{{{\mathrm{r}}}}\left( T \right)$$

with the solid conductivity λ_s_, the effective thermal conductivity of the gas phase λ_g_, and the radiative conductivity λ_r_ [21]. The solid, gas phase and radiative conductivity are determined by the porosity, pore structure, chemical composition and morphology of the aerogel, however in different ways.

In the context of diffusive radiative heat transfer, the specimen whose thermal conductivity is to be determined needs to be optically thick, i.e. the mean free path for photons within the relevant wavelength range must be significantly smaller than the specimen dimensions. Achieving optical thickness is possible through a combination of high mass density and/or high infrared extinction [22]. The radiative conductivity for optically thick aerogels is given by [23]:

3 $$\lambda _{{{\mathrm{r}}}}\left( T \right) = \frac{{16}}{3} \cdot \frac{{\sigma \cdot n^2 \cdot T_{{{\mathrm{r}}}}^3}}{{\rho \cdot e^ \ast \left( T \right)}}$$

with σ being the Stefan–Boltzmann constant, n the effective index of refraction, ρ the density of the aerogel, e*(T) the temperature-dependent effective specific extinction coefficient and T_r_ the mean radiative temperature. In the case of optically thin samples, the radiative heat transfer is non-local and depends on specimen dimensions, the optical properties of the boundaries of the analysis set-up and the temperature distribution within the specimen. Thermal conductivity measurements on these kind of specimens at certain conditions, e.g. in an extreme case, such as for evacuated low-density silica aerogels at elevated temperatures, can return erroneous results [24, 25].

**Fig. 1:**
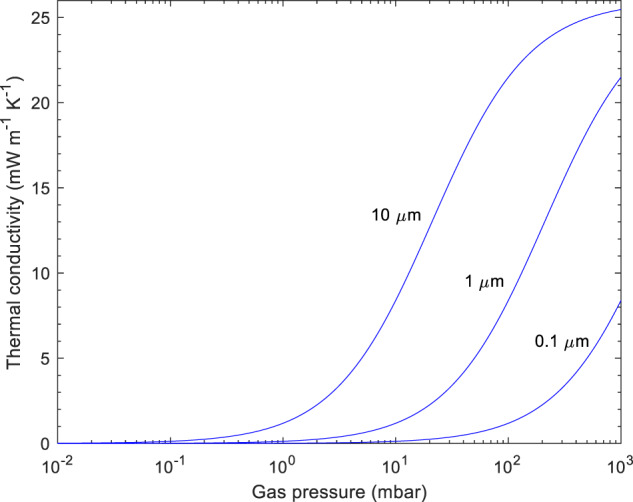
Thermal conductivity of air within a porous material as a function of gas pressure and pore diameter D (at room temperature) calculated according to Eq. (4)

The gas pressure dependence of the total effective thermal conductivity can be approximated by:

4 $$\lambda _g\left( p \right) = \frac{{\lambda _{g,0} \cdot {\Phi}}}{{\left( {1 + 2 \cdot \beta \cdot \left( {\frac{{lg,0}}{D}} \right) \cdot \left( {\frac{{p_0}}{p}} \right)} \right)}}$$

with λ_g,0_ being the gas phase thermal conductivity at a reference condition (26 mW m^−1^ K^−1^ at ambient pressure and temperature), *Φ* the porosity of the sample, β a constant with a value close to 1.5, depending on the gas and accommodation coefficient, *l*_*g,o*_ the mean free path of the gas molecules at a reference condition (70 nm for air at ambient pressure and temperature), *D* the pore diameter, *p*_*0*_ reference pressure and *p* pressure (Fig. 1) [26]. Following this equation, the Knudsen effect decreases λ_g_ at reduced gas pressures: as the mean free path of the gas molecules increases and approaches the pore diameter D, gas phase molecular collisions and their associated energy exchange become more unlikely. Conversely, at a constant pressure, λ_g_ decreases as the pore sizes become smaller and approach the mean free path of the gas molecules.

For materials with pore diameters of 5 µm and above, the Knudsen effect is insignificant at ambient pressure and temperature conditions (Fig. 1), and λ_g_ is nearly the same as for standing air, i.e. 26 mW m^−1^ K^−1^, which is a hard lower limit for the total thermal conductivity in macroporous materials. In contrast, materials with effective pore sizes of 1 µm and 100 nm do display a reduction in λ_g_ compared to bulk air, to 21 and 8 mW m^−1^ K^−1^, respectively, with a strong gas pressure dependence of the thermal conductivity at ambient pressure (Fig. 1). Therefore, it is recommended to provide also the atmospheric pressure value, present at the time of the measurement, to allow a comparison of different thermal conductivity values, which are measured at different locations and atmospheric pressure conditions [27].

The solid thermal conductivity displays a complex dependence on solid fraction, thermal conductivity of the bulk solid, phonon mean free path versus particle, neck or nanofiber cross sections, network tortuosity, and the potential alignment of solid structures, but λ_s_ is approximated here according to a more simple, general percolation model [28]:

5 $$\lambda _s\left( T \right) = \lambda _0\left( T \right)\left( {\frac{\rho }{{\rho _0}}} \right)^\alpha$$

with *λ*_0_ being the thermal conductivity of the bulk solid, ρ_0_ the density of bulk solid, and α the percolation exponent.

**Fig. 2:**
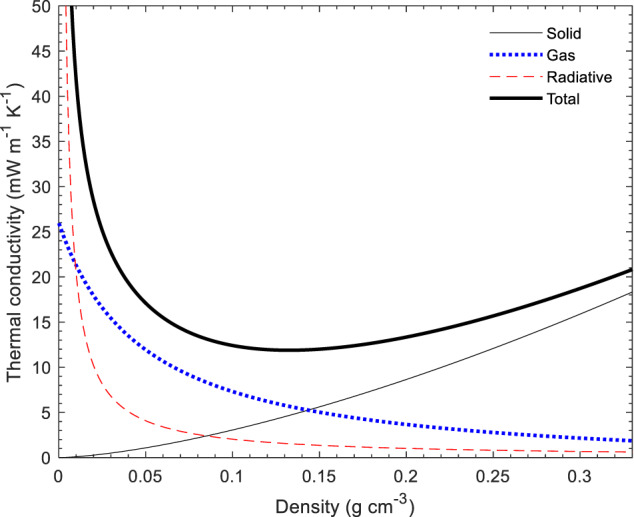
Principle profile of the temperature-dependent thermal conductivity and its components in accordance to Eqs. (2)–(5) at ambient pressure and temperature. The data were calculated by assuming the following material parameters and boundary conditions: temperature T = 300 K, pressure p = 1 bar, λ_0_ = 140 mW m^−1^ K^−1^, a temperature independent e*(T) = 40 m² kg^−1^, *n* = 1, α = 1.5, a skeletal density ρ_0_ = 1.280 g/cm^3^, and a density dependent pore size calculated from the density for a specific surface area of 400 m^2^/g using Eq. (6)

The principal profile of the total effective thermal conductivity, and its different components as a function of density in accordance to Eqs. (2)–(5), are shown in Fig. 2. A minimum of the total effective thermal conductivity as a function of density is observed, which results from opposing density dependent trends of the solid and radiative/gas phase contributions. This general picture has been proposed and was confirmed already decades ago for organic aerogels [29], and has subsequently been confirmed for many aerogel compositions [29–33].

**Fig. 3:**
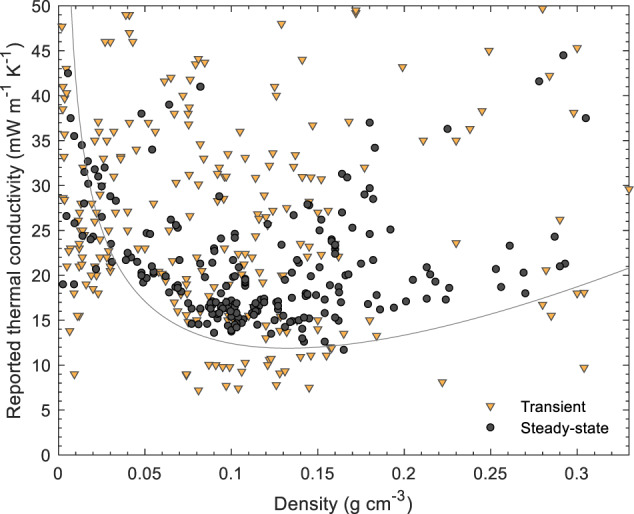
Thermal conductivity (near STP) as a function of density for biopolymer, synthetic polymer, silica and other inorganic aerogels, and their hybrids (Supplementary Table S1 for data sources). The line denotes a theoretical prediction of the thermal conductivity using the parameters from Fig. 2. Data grouped by measurement technique

**Fig. 4:**
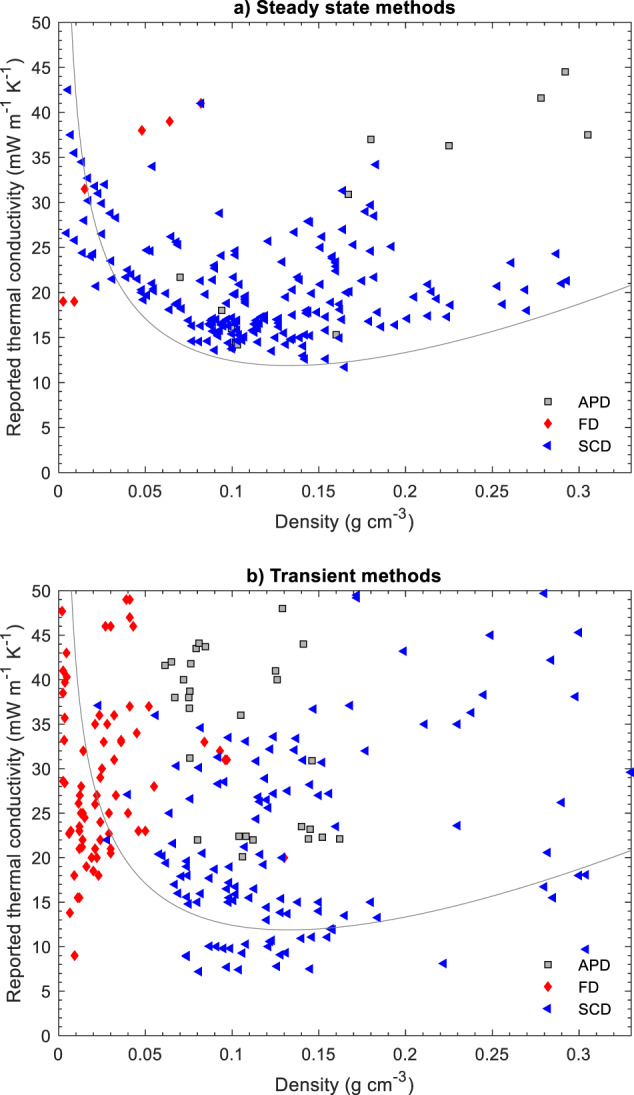
Reported thermal conductivity (near STP) as a function of density for biopolymer, synthetic polymer, silica and other inorganic aerogels, and their hybrids (Supplementary Table S1 for data sources). The line denotes a theoretical prediction of the thermal conductivity using the parameters from Fig. 2. Data grouped by drying technique (APD ambient pressure drying, FD freeze drying, SCD supercritical drying). **a** Data from steady state methods. **b** Data from transient methods

**Fig. 5:**
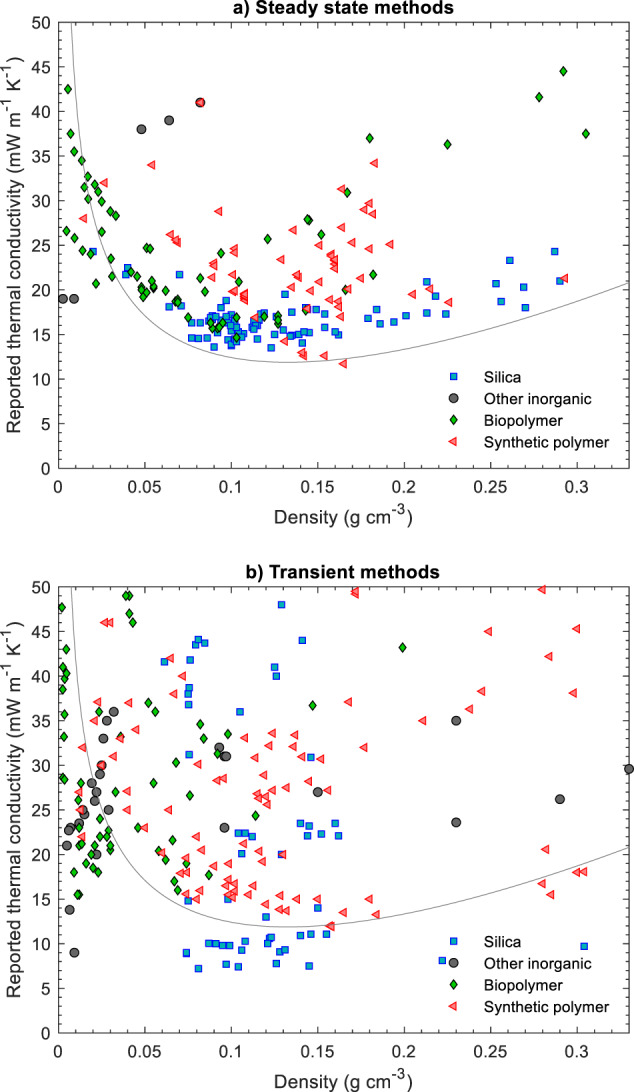
Thermal conductivity (near STP) as a function of density for biopolymer, synthetic polymer, silica and other inorganic aerogels, and their hybrids (Supplementary Table S1 for data sources). The line denotes a theoretical prediction of the thermal conductivity using the parameters from Fig. 2. Data grouped by materials system. **a** Data from steady state methods. **b** Data from transient methods

Note that although the parameters fed into Eqs. (2)–(5) depend on the material and solid skeleton and pore structure, the general shape of the density dependence of the thermal conductivity will remain the same. Experimental total thermal conductivity values well below the curve in Fig. 2 are not plausible. At very low densities (<0.020 g cm^−3^), there is no known physical mechanism to significantly reduce the gas phase conduction below that of standing air, and for most materials, also radiative contributions will be high; in this density range, the thermal conductivity of still air, 26 mW m^−1^ K^−1^, can be considered as a hard physical limit. At intermediate densities (0.050–0.200 g cm^−3^), the situation is more complex: it is, at least in theory, possible that an aerogel has the necessary high solid tortuosity and low bulk solid conductivity, small pore sizes, and high extinction coefficient, to lower the thermal conductivity to well below the curve in Fig. 2. However, based on the lack of reliable data that indicate such low values, we consider 10 mW m^−1^ K^−1^ as a practical, empirical limit, and any claim for a lower thermal conductivity would require particularly strong evidence. The rationale behind this empirical limit is more evident when the entire dataset is considered (discussion in Section 4.1, Figs. 3–5), but includes the lack of independent confirmation, the lack of steady-state data, and the lack commercial products with such performance.

Recently, Ebert et al. proposed an approximate formula that provides a lower limit of the total effective thermal conductivity of evacuated porous materials as a function of density and temperature [28]. This approach can also be applied to assess the credibility of extremely low measured values for aerogels at ambient conditions: the lower limit given has to be complemented by adding the contribution of the pore gas to these total effective thermal conductivity, considering additionally Eq. (4) and comparing these data to the experimental findings for the respective aerogel.

In summary, heat is conducted in porous materials through radiative mechanisms and gas- and solid-phase conduction. As a result, there are physical limitations to achieving high performance insulating properties and thermal superinsulation phenomena. Nevertheless, thermal conductivity values outside of these ranges are often reported (as discussed below) and hence a closer look at how thermal conductivity is measured is warranted.

## Experimental techniques to measure thermal conductivity

There are two groups of experimental methods for determining the thermal conductivity: steady state and transient methods. A detailed description of the methods and their applicability to aerogels is given elsewhere [21].

Stationary methods are based on Fourier’s law. A specimen is subjected to a temperature gradient and the temperatures at the external boundaries where this gradient is initiated, the thickness of the specimen and the heat flow through the specimen are measured. The most widely used methods in this group are the guarded-hot-plate and the heat-flow-meter method, where the specimen is placed between a ‘hot’ and ‘cold’ plate that define the temperature gradient. Under optimal measurement conditions, thermal conductivity values can be determined with a relative measurement uncertainty of less than 1% [27, 34]. It is important to note that the guarded hot plate method is an absolute method, whereas the heat flux measurement method is a relative method that requires a suitable reference sample with known thermal conductivity values, ideally in the same range as the values of the materials studied. The effective thermal conductivity is determined in the direction of the one-dimensional temperature gradient and the measured value represents an average value representative of the specimen volume between the plates. Unintended lateral heat losses, if not corrected by proper experimental design or suitable correction methods, can lead to an overestimation of the thermal conductivity value. In addition, thermal contact resistances can also lead to erroneous values.

Non-stationary methods, also known as dynamic or transient methods, for determining thermal conductivity are based on the assumption that a solution of the time-dependent equation of heat transfer is known and that an experiment can be performed in a defined and proper manner where thermal conductivity is a relevant parameter. The most used transient methods in the context of aerogel characterization are the hot-wire, hot-strip, transient-plane (hot-disc) and laser-flash method. The hot-wire, hot-strip and also the transient-plane method are absolute methods, i.e. no reference specimens are needed. In these methods, the specimen is thermally excited by a controlled heat input and the thermal response is measured and compared with the theoretical solution of the time-dependent equation of heat transfer. In most cases, the heat source is applied at the same time as the temperature sensor. In the case of the transient-plane method, the applicability of the standard evaluation procedures or measurement equipment should be tested by using reference materials with sufficiently low thermal conductivity [21, 35]. The laser-flash method allows the determination of thermal diffusivity, which is the thermal conductivity, divided by the volumetric heat capacity. The method requires either optically thick samples, such as carbon aerogels, or data correction regarding non-diffusive heat transfer. Thus, this method should not be applied to non-opacified silica aerogels. While transient methods can return accurate thermal conductivity data for aerogels, these methods are more complex with regard to the choice of the suitable measurement conditions and data analysis. Indeed, an inter-comparison of thermal conductivity measurements on PU-Aerogel showed that most of the applied transient methods, in contrast to the steady-state measurement methods, yielded unacceptably high deviations from the determined reference value of thermal conductivity [27]. Several unfavorable factors come together: typically smaller specimen geometries and measurement methods that require greater experience of the operator, particularly for the extreme material properties of aerogels. Often, this expertise may not be within the core expertise of a scientific working group who relies on standard measurement routines and analyses provided from the device manufacturers.

For all measurements, except for the laser-flash method, thermal contact resistances, e.g. uneven specimen surfaces, can influence the measurement and may result in lower or higher measurement values, respectively, depending on whether the true thermal conductivity of the sample is higher or lower than that of air. For anisotropic thermally conducting specimens, the determined thermal conductivity values depend on the method used and the orientation of the specimen within the apparatus. Thus, additional, specific details regarding the measurement conditions must be provided when reporting the thermal conductivity values.

Despite the difficulties described above, the experimental measurement of thermal conductivity remains the only way to accurately and precisely determine the thermal conductivity of aerogels. Whilst simulations of aerogel thermal conductivity can provide valuable, qualitative insights into the mechanisms of heat transport in aerogels [36–40], they are not yet sufficiently advanced to serve as a quantitative tool. One main reason for this is our lack of accurate 3D structural data on aerogels, despite recent experimental [41–43] and numerical progress in this area [36].

## Compilation of thermal conductivity data

For this paper, we have compiled 519 thermal conductivity data points from 87 different aerogel studies (Fig. 1, Supplementary Table S1). The dataset is not complete as the aerogel literature has grown too much for this to be feasible, but the compilation does provide a broad overview of the aerogel literature, including data from silica, organo-silica, ceramic, cellulose, chitosan, pectin, alginate, polyurethane, polyurea, polyimide, and resorcinol-formaldehyde aerogels, as well as their composites. The dataset incorporates recent compilations on silica [16], biopolymer [44, 45], and polyimide aerogels [46], but many additional studies have been included specifically for the current study (Table S1). Data on particulate aerogels are not included here due to the added complexity in determining their thermal conductivity, e.g. compaction and packing density of the particle/powder bed during the measurement. Note that a detailed statistical treatment is challenging, because of possibly skewed sampling of the literature during the compilation of the database, and because some studies report only a single thermal conductivity result, whereas others report a few dozen. Nevertheless, the compilation does provide a window into the state of the data quality in the aerogel literature, at least on a qualitative level.

In Fig. 3, we have grouped the data as a function of measurement technique, in Fig. 4 according to the drying technique used during aerogel preparation, and in Fig. 5 by materials system. Note, that functional dependencies can only be worked out to a limited extent from the data compilation as the measurement conditions and sample properties are too different across different studies. Even within a class of aerogels, variations in synthesis parameters can lead to significant differences in material properties. Nevertheless, certain trends and significant deviations can still be identified.

### Effect of measurement technique

In Fig. 3, the dataset is presented as a function of the method used to measure thermal conductivity. The thermal conductivity data as a function of aerogel density, as determined by steady-state methods, display a well-defined lower boundary below which only few thermal conductivity data are reported. This well-defined boundary is remarkable considering the wide variety of materials studied: pectin, cellulose, silica, organo-silica, RF, polyimide, polyurethane. The use of steady-state measurement methods tends to return more reliable values of thermal conductivity as described in the measurement section above. Due to the available experimental setup, in the most cases larger specimen dimensions, e.g. 0.2 m in diameter or (0.2 × 0.2) m², are needed to perform stationary measurements. Thus, average values for the thermal conductivity will be derived and the result is less sensitive to inhomogeneities. However, even with stationary methods, greater uncertainties and errors can occur in individual cases if the measurement is carried out improperly, for example due to parasitic heat losses or gains at the specimen edges. The steady-state data (Fig. 3) show the expected minimum in thermal conductivity at ambient conditions at densities between 0.080 and 0.180 g/cm^3^, depending on the specific material/study [29–33], as well as the expected significant increase in thermal conductivity at lower densities due to the increase in radiative and gas-phase contributions (Fig. 2). The relatively high densities at the thermal conductivity minimum – for comparison, mineral wool and polymer foam insulation products typically have densities below 0.050 g/cm^3^ - are necessary to ensure an adequate mass is present to divide the pore volume into sufficiently small pores where the Knudsen effect can effectively reduce thermal conductivity (Eq. (4)).

In contrast to the relatively consistent data from steady state methods, the data generated by transient methods scatter much more widely, without a clear dependence on density (Fig. 3). A significant fraction of this scatter may be due to the different materials investigated. Nevertheless, nearly all of the physically impossible results, e.g. values below 26 mW m^−1^ K^−1^ for macroporous, low density freeze dried foams (Fig. 4b), or empirically improbable data, e.g. values below 10 mW m^−1^ K^−1^ for any type of aerogel, were measured with transient methods. No commercial products declare thermal conductivities below 12–15 mW m^−1^ K^−1^, yet there are some studies that present data as low as 9 mW m^−1^ K^−1^. If real, such a performance would be of extreme commercial interest, but these reports have not been reproduced, and none of these materials are available on the market. Hence, claims of thermal conductivity values below 10 mW m^−1^ K^−1^, and possibly also below 12 mW m^−1^ K^−1^ are most likely incorrect. The abundance of improbable thermal conductivity data from transient methods does not imply that all results from such methods are questionable. In fact, many of the seminal papers on aerogel thermal conductivity were based on transient data (hot-wire) [29, 33]. If carried out properly, transient measurements can provide accurate thermal conductivity data in a convenient and fast manner. However, the abundance of so many questionable data from transient methods does imply that additional skepticism is warranted when evaluation results from transient methods. Particularly the last decade has seen a rapid proliferation of highly unlikely thermal conductivity values from hot-disk measurements (Supplementary Table S1).

Irrespective of the selected method, there is a strong burden of proof on any study/material that claims a performance beyond physically expected values, i.e. much lower than expected thermal conductivities for a given density or pore structure: “Extraordinary claims require extraordinary evidence.”. This evidence has to be shown by a detailed description of the measurement equipment, the measurement conditions (temperature, atmosphere, atmospheric pressure, humidity) and a detailed uncertainty assessment according to the GUM (Evaluation of measurement data - Guide to the Expression of Uncertainty in Measurement [47]). Available recommendations should be followed [21] and/or measurement routines tested by measuring aerogels with a validated thermal conductivity [27]. Following points are also important:

- In the ideal case, the measured values of total effective thermal conductivity should accurately represent the true values within the associated uncertainties. This should be the case for all optically thick aerogel specimens.
- For optically thin aerogels, e.g. ultra-low density silica aerogels, which are measured with a stationary guarded hot plate method, the indication of a thermal conductivity value is questionable.
- The measurement uncertainties for thermal conductivity values depend on both the experimental method and on to what extent the specimen is suitable to the specific measurement equipment, e.g. in respect to the size of the specimen or external surface properties.
- Critically important is the experience of the operator performing the thermal conductivity measurements and their awareness that specimens with expected low values of thermal conductivity are investigated, which in some cases are outside the specifications of the instrument, calibration or standards.
- According to the guidelines from the IEA EBC Annex 65 subtask 2 for thermal conductivity measurements on superinsulation materials by means of the guarder hop-plate and heat flow meter method, a minimum temperature difference of 15 K is recommended [48].

### Effect of drying technique

In Fig. 4, the same dataset as for Fig. 3 is presented, but now grouped as a function of the drying technique used to prepare the aerogels.

Supercritical drying (SCD) is the gold standard to preserve the gel’s delicate structures during drying [18, 49], and the resulting aerogels typically have the highest surface areas and highest fraction of mesopore volume favoring suppression of gas phase heat transport at ambient conditions (Fig. 1). Aerogels produced by supercritical drying are commercially available, and fiber-reinforced silica aerogel blankets are by far the most successful aerogel product in the market. The steady-state thermal conductivity data (Fig. 4a) of the SCD aerogels define a clear trend and the data are in line with the physical boundary conditions described above. The transient data (Fig. 4b) for the SCD aerogels are much more scattered and include some unrealistically low thermal conductivity data.

Ambient pressure drying (APD) often leads to strong pore collapse, but can maintain or recover a significant fraction of mesopores in specific cases, particularly for silica aerogel [50]. Often, only particulate materials and composites can be produced, but the performance sometimes rivals that of SCD materials [50–55] and silica aerogel granulate, powders and blankets produced by APD are available commercially. Unfortunately, insufficient data are available for APD aerogels to derive meaningful conclusion for this data compilation.

Finally, freeze drying (FD) leads to the formation of large, secondary pores due to ice crystal growth, most often up to tens of micrometers in diameter. While this technique enables the production of low-density materials with interesting mechanical properties with structures that may be of interest for non-insulation applications, the freeze drying process, particularly from water, does remove most of the mesopore volume and there is no physical mechanism that can reduce the thermal conductivity below that of standing air in such macroporous materials. Nevertheless, many studies using transient methods, and some using steady state methods, report extremely low thermal conductivities for macroporous FD materials, well below that of standing air or the best performing conventional insulation materials, and often even at densities much below the thermal conductivity minimum for the more homogenous, mesoporous SCD aerogels (Fig. 3).

### Effect of aerogel composition

Finally, in Fig. 5, we have grouped the data per material class, and separated depending on the measurement technique. The data derived from steady state methods (Fig. 5a) display remarkably narrow trends, particularly for biopolymer and silica aerogels. The data for synthetic polymers are more scattered, most likely due to the significant variations in polymer type (resorcinol-formaldehyde, polyurethane, polyurea, polyimide), the inclusion of composites in the dataset, and the different microstructures. High performance materials with credible thermal conductivity data (<20 mW m^−1^ K^−1^ at 0.080 to 0.120 g cm^−1^) have been reported for all three major material classes (silica, biopolymers and synthetic polymers). Note that for a given morphology, polymeric aerogels should yield the lowest thermal conductivities due to their lowest intrinsic solid phase conductivity, and the compiled steady state data hint at this effect, with the overall lowest reported values for synthetic polymers and a minimum at somewhat higher densities compared to silica aerogels. Careful seminal studies with transient methods also observed the same [33].

The overall dataset from transient methods (Fig. 5b) scatters wildly, with unexpectedly low thermal conductivity results for most classes of materials, e.g. values below 10 mW m^−1^ K^−1^ for silica aerogels and values well below that of standing air for biopolymer and non-silica inorganic aerogels at densities where the gas phase conduction cannot be suppressed substantially (<0.030 g cm^−1^).

## Discussion

We want to stress that physically improbable thermal conductivity data are not limited to low-quality journals or authors from lower-ranked institutes. Unlikely data are reported from some the most renowned universities and from all over the world (Asia, North America, Europe and Africa) and published in many of the most important journals in field, including some with impact factors above 60. Hence, the problem of unreliable thermal conductivity data is not something in the periphery, but threatens the very core of the aerogel science field. In many cases, the anomalously low thermal conductivity results are also the key result around which the paper is written, and the supposed superinsulation nature of the materials is often highlighted in the title.

The first reason for the preponderance of erroneous thermal conductivity data are certainly the analytical challenges to measure thermal conductivity accurately, particularly for the small samples that are typically available in research and early stage R&D (see above). Aside from the physical limitations of the different analytical techniques, there are also organizational challenges related to access to instruments, expertise of operators and the suitability of calibrations using materials that differ substantially from the aerogels in question. Often, aerogel research is driven by research groups with a strength in chemistry and materials synthesis, rather than characterization. While these groups often make important, highly original contributions to the aerogel field on the synthesis side, they sometimes lack the experience with aerogel characterization to spot potential issues with their thermal conductivity analysis. Cooperation between materials and methods experts could significantly improve the reliability of the data.

A compounding factor of why physically improbable thermal conductivities go undetected are analytical difficulties with the pore size determination [56, 57]. No analytical technique is able to quantify the pore size distribution over the length scales relevant for most aerogels, from single nanometers to tens of micrometers. The most common technique, nitrogen sorption analysis with BJH or NLDFT analysis to convert the sorption isotherms into pore size distributions, is only sensitive to pore sizes smaller than 50–100 nm and larger pores go undetected. In addition, deformation of the sample during analysis may result in significantly underestimated pore sizes [58, 59]. Hence, gas sorption analysis returns, by definition, an average pore size that is in or close to the mesoporous range (2–50 nm). When such artificially low pore sizes are reported uncritically, it may appear that the Knudsen effect is expected to be significant (Eq. (4)), and it may thus not be immediately obvious just how improbable the anomalously low thermal conductivity data really are. Thus, for a first check of average pore size *D* the following relationship assuming cylindrical pores can be used:

6 $$D = \frac{{4V_P}}{{{{\mathrm{S}}}}}\,with\,V_{{{\mathrm{p}}}} = \frac{1}{{\rho _{bulk}}} - \frac{1}{{\rho _{{{{\mathrm{skeleton}}}}}}}$$

Here V_p_ is the total mass specific pore volume present, S the specific surface area, as detected e.g. by N_2_ adsorption, and ρ_bulk_ and ρ_skeleton_ are the macroscopic bulk density and the density of the non-porous skeleton, respectively. For pore size values above 100 nm and 1 µ, the contribution due to the gas phase heat transport alone already exceeds 8 and 21 mW m^−1^ K^−1^, respectively (Fig. 1).

Unfortunately, authors, reviewers, editors and readers sometimes do not have sufficient expertise to recognize inaccurate or highly improbable thermal conductivity results, for example because they are more versed in aerogel synthesis than characterization. In addition, authors are strongly incentivized to not question the supposedly very low thermal conductivity of their materials: better performance increases outside interest, eases publication in higher profile journals, and may help or be perceived to help researchers with advancing their careers, at least in the short to medium term.

It is important to note that erroneous thermal conductivity data are not limited to cases where physically improbable values are reported, those cases are just easier to spot and point out in a data compilation. Indeed, it is almost certain that a significant fraction of the data that appear reasonable and within the physically allowed region of Fig. 2 are also incorrect, because many of the analytical challenges and incentives remain the same. For example, superinsulating thermal conductivity values are sometimes reported at densities that may appear reasonable, but a closer look into the materials indicates that the microstructure is macroporous.

The problem with the reliability of thermal conductivity data is not limited to the aerogel scientific field, but of general concern in research on thermal insulation materials and even in the performance of thermal insulation products on the market. Conventional thermal insulation materials are characterized by air-filled pores in the micro- to millimeter range, and thus inevitably have a thermal conductivity in excess of the thermal conductivity of standing air (~26 mW m^−1^ K^−1^). Nevertheless, the scientific literature contains numerous reports on conventional insulation with lower values, e.g. biomass-based materials such as cotton fibers, hemp fibers or rice husk. In these studies, both transient and steady state methods are used, and the obviously incorrect data are more likely due to incorrect execution of the measurement or conditioning of the samples, rather than a specific method. Obviously, this basic consideration only reveals physically impossible thermal conductivity data when the values are very low, and it is likely that many incorrect data remain undetected for higher, seemingly more plausible thermal conductivity values. For example, reports of thermal conductivities in the range of 27–32 mW m^−1^ K^−1^ are common for biomass-based insulation, which, while not physically impossible, are nevertheless very unlikely given the solid and radiative contributions on top of the gas phase conduction [60] and considering that no biomass-based thermal insulators with a thermal conductivity below 36 mW m^−1^ K^−1^ are commercially available.

Commercial thermal insulation materials are more regulated with standardized methodologies (e.g. EN 12667) and large quantities of larger samples are available. However, different insulation products often require different approaches to yield useful and representative thermal conductivity data. For example, silica aerogel blankets are compressible under the load of a large guarded hot plate device leading to lower thermal conductivities due to the reduction of pore volume between the aerogel grains during the measurement. This issue was recently addressed by the standard ISO 22482:2021, which requires the thermal conductivity measurement at the measured thickness of the sample, i.e. with a mechanical support within the guarded hot plate device. In other cases, standards and regulation seem not to result in measurement and reporting conditions that are representative of the intended application scenario. For example, the thermal conductivity of insulating bricks is often based on dry bricks, to which a theoretical factor of a few percent is added to account for moisture. In some cases, this factor is omitted altogether and only the dry thermal conductivity is reported. This leads to measured values up to 35% higher than the declared values, when measured at standard conditions for insulation (50% relative humidity), e.g. 91 mW m^−1^ K^−1^ for a declaration of 70 mW m^−1^ K^−1^ [61]. In these cases, the measurement and/or declaration procedure is clearly not representative of the actual application in buildings – where the material is not in a dry state – leading to an overestimation of the thermal performance of the entire building.

These examples indicate that also in research on conventional insulation materials, as well as in the insulation material market, experience and a critical mind are necessary to measure, report and evaluate thermal conductivity. For commercial insulation products and in the building applications themselves, a more rigorous performance control is necessary to close the gap between reported and actual performance [62].

## Call to action

Our goal in writing this paper is not to call out individual researchers, but rather to encourage skepticism among readers, editors and reviewers, and most of all among the aerogel researchers and authors themselves. Researchers should have a comprehensive understanding of the method they select to measure thermal conductivity, and be aware of its limitations when applied to their specific materials and sample size. Both steady-state and transient methods can return accurate thermal conductivity values for aerogels, but in practice, transient methods appear particularly prone to return spurious results. In each case, for both methods, instruments should be calibrated and their accuracy validated using samples with similar properties and microstructures. Even then, instrument read-outs should not be taken at face value but treated with caution, particularly if the results appear too good to be true: “extraordinary claims require extraordinary evidence”. Whilst the primary responsibility clearly lies with the authors, reviewers and editors are advised to question the accuracy of the data and insist that the authors provide detailed calibration and validation data on their measurement technique. Finally, until the situation improves, readers cannot simply trust any aerogel thermal conductivity data in the literature. Instead, they should assess whether the data were acquired with appropriate methods and evaluate how feasible a result is based on the physical boundary conditions determined by the density, microstructure and pore sizes of the materials in question.

On a more positive note, many aerogels do have record-breaking, ultra-low thermal conductivities and thermal insulation remains the most important and unique selling point in the market, with new emerging opportunities in battery thermal runaway protection for electric mobility. The rate of progress in the field is higher than ever, and increased quality standards in measuring, reporting and interpreting thermal conductivity data will ensure that this rate of progress can be maintained moving forward.

### Supplementary information

Supplementary Information

## Data Availability

CAE is willing to provide polyurethane aerogel samples, which are the same material than used in a recent inter-comparison study [23], with an individually determined value of thermal conductivity at nominal cost (https://en.cae-zerocarbon.de/Thermal-Reference-Material.html).

## References

[1] Aegerter MA, Leventis N, Koebel MM (2011). Aerogels handbook.

[2] Hüsing N, Schubert U (1998). Aerogels-airy materials: chemistry, structure, and properties. Angew Chem Int Ed.

[3] Ratke L, Gurikov P (2021) The chemistry and physics of aerogels: synthesis, processing, and properties. Cambridge University Press, Cambridge, UK

[4] Baetens R, Jelle BP, Gustavsen A (2011). Aerogel insulation for building applications: a state-of-the-art review. Energy Build.

[5] Koebel MM, Rigacci A, Achard P (2012). Aerogel-based thermal. Superinsulation.

[6] Kistler S, Caldwell A (1934). Thermal conductivity of silica aerogel. Ind Eng Chem.

[7] Garcia-Gonzalez CA, Alnaief M, Smirnova I (2011). Polysaccharide-based aerogels - promising biodegradable carriers for drug delivery systems. Carbohydr Polym.

[8] Smirnova I, Suttiruengwong S, Arlt W (2004). Feasibility study of hydrophilic and hydrophobic silica aerogels as drug delivery systems. J Non Crystalline Solids.

[9] Wei Z (2021). Nanocellulose based hydrogel or aerogel scaffolds for tissue engineering. Cellulose.

[10] Maleki H, Hüsing N (2018). Current status, opportunities and challenges in catalytic and photocatalytic applications of aerogels: environmental protection aspects. Appl Catal B Environ.

[11] Budtova T (2023). Acoustic properties of aerogels: current status and prospects.. Adv Eng Mater.

[12] Mazrouei-Sebdani Z (2021). A review on silica aerogel-based materials for acoustic applications. J Non Crystalline Solids.

[13] Sun J (2023). Aerogel-based solar-powered water production from atmosphere and ocean: a review. Mater Sci Eng R Rep.

[14] Maleki H (2016). Recent advances in aerogels for environmental remediation applications: a review. Chem Eng J.

[15] IDTechEX, Collins R (2021) Aerogel 2021-2031: technologies, markets and players

[16] Li C et al. (2023) Silica aerogels: from materials research to industrial applications. Int Mater Rev 68:862–900

[17] Koebel MM, Wernery J, Malfait WJ (2017). Energy in buildings - policy, materials and solutions. MRS Energy Sustain.

[18] Kistler SS (1931). Coherent expanded aerogels and jellies. Nature.

[19] Vareda JP, Lamy-Mendes A, Durães L (2018). A reconsideration on the definition of the term aerogel based on current drying trends. Microporous Mesoporous Mater.

[20] Swimm K (2017). Coupling of gaseous and solid thermal conduction in porous solids. J Non Crystalline Solids.

[21] Ebert H-P (2011) Thermal properties of aerogels. In: Aerogels handbook. Springer, Berlin, Germany, p. 537–564

[22] Fricke J (1990). Opaque silica aerogel insulations as substitutes for polyurethane (PU) foams. Therm Conductivity.

[23] Siegel R (2001) Thermal radiation heat transfer. CRC Press, Boca Raton, USA

[24] Ebert H-P, Fricke J (1998). Influence of radiative transport on hot-wire thermal conductivity measurements. High Temp High Press.

[25] Heinemann U (1993) Wärmetransport in semitransparenten nichtgrauen Medien am Beispiel von SiO2-Aerogelen

[26] Reichenauer G, Heinemann U, Ebert H-P (2007). Relationship between pore size and the gas pressure dependence of the gaseous thermal conductivity. Colloids Surf A Physicochemical Eng Asp.

[27] Ebert H-P (2021). Intercomparison of thermal conductivity measurements on a nanoporous organic aerogel. Int J Thermophys.

[28] Ebert H-P, Manara J, Reichenauer G (2023). Limits of thermal insulations: heat transfer within evacuated porous high-performance insulations. Int J Thermophys.

[29] Lu X (1992). Thermal conductivity of monolithic organic aerogels. Science.

[30] Sivaraman D et al. (2022) Superinsulating nanocellulose aerogels: effect of density and nanofiber alignment. Carbohydrate Polym 292:119675 10.1016/j.carbpol.2022.119675 35725170

[31] Wong JC (2015). Mechanical and thermal properties of nanofibrillated cellulose reinforced silica aerogel composites. Microporous Mesoporous Mater.

[32] Zhu Z (2017). Superinsulating polyisocyanate based aerogels: a targeted search for the optimum solvent system. ACS Appl Mater Interfaces.

[33] Lu X (1992). Thermal transport in organic and opacified silica monolithic aerogels. J Non Crystalline Solids.

[34] Hemminger W, Jugel R (1985). A guarded hot-plate apparatus for thermal conductivity measurements over the temperature range− 75 to 200‡ C. Int J Thermophys.

[35] Zhang H (2013). A numerical study on the influence of insulating layer of the hot disk sensor on the thermal conductivity measuring accuracy. Prog Comput Fluid Dyn Int J.

[36] Yeo J, Liu Z, Ng TY (2020) Silica aerogels: a review of molecular dynamics modelling and characterization of the structural, thermal, and mechanical properties. In: Handbook of materials modeling: applications: current and emerging materials. Springer, Berlin, Germany, p. 1575–1595

[37] Patil SP (2021). Mechanical modeling and simulation of aerogels: A review. Ceram Int.

[38] Liu J (2022). Microscopic revelation of the solid–gas coupling and Knudsen effect on the thermal conductivity of silica aerogel with inter-connected pores. Sci Rep..

[39] Swimm K (2017). Impact of thermal coupling effects on the effective thermal conductivity of aerogels. J Sol-Gel Sci Technol.

[40] Iswar S et al. (2021) Dense and strong, but superinsulating silica aerogel. Acta Materialia 213:116959

[41] Roiban L (2016). Advanced three dimensional characterization of silica-based ultraporous materials. RSC Adv.

[42] Chal B (2021). 3D multi-scale quantification of industrially relevant ultra-porous silicas by low-dose electron tomography combined with SANS. J Non Crystalline Solids.

[43] Reichenauer G (2023) Structural characterization of aerogels. In: Springer handbook of aerogels. Springer, Berlin, Germany, p. 151–195

[44] Guerrero-Alburquerque N (2020). Strong, machinable, and insulating chitosan–urea aerogels: toward ambient pressure drying of biopolymer aerogel monoliths. ACS Appl Mater interfaces.

[45] Zhao S (2018). Biopolymer aerogels and foams: chemistry, properties and applications. Angew Chem Int Ed.

[46] Kantor Z (2022). Heterogeneous silica-polyimide aerogel-in-aerogel nanocomposites. Chem Eng J.

[47] Metrology JCFGI (2008). Evaluation of measurement data—Guide to the expression of uncertainty in measurement. JCGM.

[48] Holm A et al. (2020) Annex 65, long-term performance of super-insulating-materials in building components and systems. Report of subtask II: scientific information for standardization bodies dealing with hygro-thermo-mechanical properties and ageing, IEA, Paris, France

[49] Tewari PH, Hunt AJ, Lofftus KD (1985). Ambient-temperature supercritical drying of transparent silica aerogels. Mat Lett.

[50] Prakash SS (1995). Silica aerogel films prepared at ambient pressure by using surface derivatization to induce reversible drying shrinkage. Nature.

[51] Martinez RG (2016). Thermal assessment of ambient pressure dried silica aerogel composite boards at laboratory and field scale. Energy Build.

[52] Shimizu T (2016). Transparent, highly insulating polyethyl-and polyvinylsilsesquioxane aerogels: mechanical improvements by vulcanization for ambient pressure drying. Chem Mater.

[53] Huber L (2017). Fast and minimal-solvent production of superinsulating silica aerogel granulate. Angew Chem Int Ed.

[54] Hayase G (2014). The thermal conductivity of polymethylsilsesquioxane aerogels and xerogels with varied pore sizes for practical application as thermal superinsulators. J Mater Chem A.

[55] Kanamori K (2007). New transparent methylsilsesquioxane aerogels and xerogels with improved mechanical properties. Adv Mater.

[56] Horvat G (2022). A brief evaluation of pore structure determination for bioaerogels. Gels.

[57] Reichenauer G, Aegerter MA, Leventis N, Koebel MM (2011). Structural Characterization of Aerogels. Aerogels handbook.

[58] Reichenauer G, Scherer G (2000). Nitrogen adsorption in compliant materials. J Non Crystalline Solids.

[59] Reichenauer G, Scherer G (2001). Nitrogen sorption in aerogels. J Non Crystalline Solids.

[60] Smith DS (2013). Thermal conductivity of porous materials. J Mater Res.

[61] Wernery J (2017). Aerobrick—an aerogel-filled insulating brick. Energy Procedia.

[62] De Wilde P (2014). The gap between predicted and measured energy performance of buildings: a framework for investigation. Autom Constr.

