# Supplementary material for: The poor reliability of thermal conductivity data in the aerogel literature: a call to action!
Source: J Solgel Sci Technol. 2024 Jan 3;109(2):569–79. doi: 10.1007/s10971-023-06282-9 (PMC10896818; doi:10.1007/s10971-023-06282-9)
Supplement: Supplementary file 1 — Supplementary Information [file 10971_2023_6282_MOESM1_ESM.pdf]

Supporting Information

The poor reliability of thermal conductivity data in the aerogel literature: a call to action!

Wim J. Malfait, Hans-Peter Ebert, Samuel Brunner, Jannis Wernery, Sandra Galmarini, Shanyu Zhao, Gudrun Reichenauer
wim.malfait@empa.ch, hans-peter.ebert@cae-zerocarbon.de

Table S1. Compilation of aerogel thermal conductivity data.

	Material	Density	λ	Drying	Measurement device	Measurement technique	Reference	Link
		g/cm^3	mW/(m K)					
**1**	Silica	0.106	20.1	APD	Hot Disk TPS 2500	Transient	Shao, Z. et al. (2015). Ambient pressure dried shape-controllable sodium silicate based composite silica aerogel monoliths. *Materials Chemistry and Physics*, *162*, 346-353.	https://www.sciencedirect.com/science/article/pii/S0254058415301371
		0.108	22.4					
		0.104	22.4					
		0.146	30.9					
**2**	Silica GO	0.074	8.9	SCD	Hot Disk TPS 2500	Transient	Lei, Y.et al. (2017). Enhancements of thermal insulation and mechanical property of silica aerogel monoliths by mixing graphene oxide. *Materials Chemistry and Physics*, *187*, 183-190.	https://www.sciencedirect.com/science/article/pii/S0254058416308975
		0.081	7.2					
		0.097	7.7					
		0.145	7.5					
**3**	Organosilica	0.125	41.0	APD	HotDisk TPS2500	Transient	Yun, S.et al. (2017). Facile synthesis of large-sized monolithic methyltrimethoxysilane-based silica aerogel via ambient pressure drying. *Journal of Sol-Gel Science and Technology*, *83*, 53-63.	https://link.springer.com/article/10.1007/s10971-017-4377-0#Sec6
		0.075	38.0					
		0.105	36.0					
		0.141	44.0					
**4**	Silica/Alumina	0.074	9.0	SCD	HotDisk TPS2500	Transient	Lei, Y.et al. (2017). Improvement of thermal insulation performance of silica aerogels by Al2O3 powders doping. *Ceramics International*, *43* (14), 10799-10804.	https://www.sciencedirect.com/science/article/pii/S027288421730901X
		0.104	7.4					
		0.126	7.8					
		0.222	8.1					
		0.304	9.7					
**5**	Organosilica	0.076	38.7	APD	HotDisk TPS2500	Transient	Lamy-Mendes, A.,et al. (2019). Polysilsesquioxane-based silica aerogel monoliths with embedded CNTs. Microporous and Mesoporous Materials, 288, 109575.	https://www.sciencedirect.com/science/article/pii/S1387181119304184
		0.061	41.6					
		0.079	43.5					
		0.076	41.8					
		0.075	36.8					
		0.076	31.2					
		0.081	44.1					
		0.085	43.7					
**6**	Silica	0.230	60.0	SCD	Hot Wire	Transient	Wagh, P. B.et al. (1999). Comparison of some physical properties of silica aerogel monoliths synthesized by different precursors. *Materials chemistry and physics*, *57* (3), 214-218.	https://www.sciencedirect.com/science/article/pii/S025405849800217X
		0.129	20.0					
		0.098	15.0					
**7**	Silica	0.077	16.3	SCD	Home-built guarded hotplate	Steady-state	Iswar, S.et al. (2021). Dense and strong, but superinsulating silica aerogel. *Acta Materialia*, *213*, 116959.	https://www.sciencedirect.com/science/article/pii/S1359645421003396
		0.092	15.2					
		0.115	14.5					
		0.194	16.4					
		0.224	17.3					
		0.256	18.7					
		0.269	20.3					
		0.071	18.2					
		0.089	16.7					
		0.112	15.8					
		0.184	17.8					
		0.218	19.3					
		0.261	23.3					
**8**	Silica/Organosilica	0.087	16.5	SCD	Home-built guarded hotplate	Steady-state	Iswar, S.et al. (2018). Reinforced and superinsulating silica aerogel through in situ cross-linking with silane terminated prepolymers. *Acta Materialia*, *147*, 322-328.	https://www.sciencedirect.com/science/article/pii/S1359645418300624
		0.100	15.4					
		0.107	15.1					
		0.104	15.3					
		0.106	14.7					
		1.119	14.8					
		0.125	15.0					
		0.139	15.0					
		0.145	15.2					
		0.154	15.8					
		0.179	16.8					
		0.089	17.1					
		0.100	17.2					
		0.126	17.0					
		0.117	17.3					
		0.149	17.8					
		0.213	20.9					
		0.094	16.4					
		0.102	16.8					
		0.116	16.6					
		0.119	17.4					
		0.144	17.8					
		0.102	16.5					
		0.103	15.8					
		0.094	16.6					
		0.115	16.0					
		0.113	16.5					
		0.135	17.5					
		0.143	18.0					
		0.097	17.0					
		0.088	16.9					
		0.091	17.0					
		0.098	16.3					

**9**	Resorcinol-Formaldehyde	0.157	12.0	SCD	Home-built hotwire	Transient	Lu, X. M. C. A. S.et al. (1992). Thermal conductivity of monolithic organic aerogels. *Science* , *255* (5047), 971-972.	https://www.science.org/doi/epdf/10.1126/science.255.5047.971
		0.080	15.0					
		0.300	18.0					
**10**	Silica	0.075	14.8	SCD	Home-built hotwire	Transient	Lu, X.et al. (1992). Thermal transport in organic and opacified silica monolithic aerogels. *Journal of non-crystalline solids* , *145* , 207-210.	https://www.sciencedirect.com/science/article/pii/S0022309305804570
		0.150	14.0					
		0.120	13.0					
**11**	Silica	0.081	14.6	SCD	Home-built guarded hotplate	Steady-state	Wong, J. C.et al. (2014). Mechanical properties of monolithic silica aerogels made from polyethoxydisiloxanes. *Microporous and mesoporous materials* , *183* , 23-29.	https://www.sciencedirect.com/science/article/pii/S1387181113004174
		0.100	13.8					
		0.123	13.5					
		0.141	14.1					
		0.162	15.0					
		0.201	17.1					
		0.253	20.7					
		0.287	24.3					
**12**	Polyurethane	0.183	34.2	SCD	Home-built guarded hotplate	Steady-state	Zhu, Z.et al. (2017). Superinsulating polyisocyanate based aerogels: a targeted search for the optimum solvent system. *ACS applied materials & interfaces* , *9* (21), 18222-18230.	https://pubs.acs.org/doi/pdf/10.1021/acsami.7b03344
		0.164	31.3					
		0.192	25.1					
		0.215	20.1					
		0.354	33.5					
		0.344	41.3					
		0.414	34.3					
		0.226	18.6					
		0.205	19.5					
		0.293	21.3					
		0.158	23.8					
**13**	Polurea/polyurethane	0.083	20.5	SCD	Step change/diffusivity	Transient	Lee, J. K. et al. (2009). Polyurea based aerogel for a high performance thermal insulation material. *Journal of sol-gel Science and Technology* , *49* , 209-220.	https://link.springer.com/article/10.1007/s10971-008-1861-6
		0.090	18.7					
		0.097	16.5					
		0.110	15.5					
		0.150	15.0					
		0.165	13.5					
		0.285	15.5					
		0.074	18.0					
		0.071	17.9					
		0.098	17.2					
		0.098	15.5					
		0.102	15.9					
		0.128	15.4					
		0.101	15.2					
		0.113	16.5					
		0.132	13.7					
		0.099	19.0					
		0.118	19.2					
		0.107	21.2					
		0.116	20.4					
		0.096	28.5					
		0.115	26.8					
**14**	Graphene oxide	0.009	9.0	FD	TPS	Transient	Peng, Q. et al. (2017). Superlight, mechanically flexible, thermally superinsulating, and antifrosting anisotropic nanocomposite foam based on hierarchical graphene oxide assembly. *ACS AMI* , *9* (50), 44010-44017	https://pubs.acs.org/doi/full/10.1021/acsami.7b14604
**15**	Cellulose	0.013	28.0	FD	Hot-strip device	Transient	Jiménez-Saelices, C. et al. (2017). Spray freeze-dried nanofibrillated cellulose aerogels with thermal superinsulating properties. *Carbohydrate polymers* , *157* , 105-113.	https://www.sciencedirect.com/science/article/pii/S0144861716311249
		0.024	24.0					
		0.033	27.0					
		0.012	21.0					
		0.023	18.0					
		0.030	20.5					
**16**	Polyimide/Cellulose	0.046	23.0	FD	Hot Disk TPS 2500	Transient	Zhang, X.et al. (2020). Bidirectional anisotropic polyimide/bacterial cellulose aerogels by freeze-drying for super-thermal insulation. *Chemical Engineering Journal* , *385* , 123963.	https://www.sciencedirect.com/science/article/pii/S1385894719333789
**17**	Celllose	0.012	23.0	FD	Hot-strip device	Transient	Jiménez-Saelices, C.et al. (2018). Thermal superinsulating materials made from nanofibrillated cellulose-stabilized pickering emulsions. *ACS applied materials & interfaces* , *10* (18), 16193-16202.	https://pubs.acs.org/doi/full/10.1021/acsami.8b02418
		0.014	21.2					
		0.020	18.5					
		0.024	22.0					
		0.029	22.7					
**18**	Ceramic Fibers	0.007	23.0	FD	Hot Disk TPS 2000	Transient	Liu, F. et al. (2023). Ultralight Ceramic Fiber Aerogel for High-Temperature Thermal Superinsulation. *Nanomaterials* , *13* (8), 1305.	https://www.mdpi.com/2079-4991/13/8/1305
		0.006	22.7					
		0.012	23.5					
		0.015	24.5					
		0.021	26.0					
**19**	Silicon carbide	0.007	13.8	FD	LFA467 Laser Flash	Transient	Su, L.et al. (2020). Anisotropic and hierarchical SiC@ SiO2 nanowire aerogel with exceptional stiffness and stability for thermal superinsulation. *Science advances* , *6* (26), eaay6689.	https://www.science.org/doi/epdf/10.1126/sciadv.aay6689
**20**	Boron nitride	0.032	36.0	FD	Hot Disk TPS 2500	Transient	Adegun, M. H., et al. (2023). Anisotropic thermally superinsulating boron nitride composite aerogel for building thermal management. *Composites Part A: Applied Science and Manufacturing* , *169* , 107522.	https://www.sciencedirect.com/science/article/pii/S1359835X23000982
		0.022	20.0					
		0.029	25.0					
		0.093	32.0					
		0.096	31.0					
		0.097	31.0					
**21**	Cellulose	0.021	21.0	FD	Hot Disk TPS 2500	Transient	Song, M.,et al. (2020). Flexible and super thermal insulating cellulose nanofibril/emulsion composite aerogel with quasi-closed pores. *ACS Applied Materials & Interfaces* , *12* (40), 45363-45372.	https://pubs.acs.org/doi/full/10.1021/acsami.0c14091
		0.019	20.0					
		0.016	19.0					
		0.012	15.5					
		0.011	15.5					
**22**	Cellulose	0.008	28.3	SCD	Unclear	Unclear	Abraham, E.et al. (2023). Highly transparent silanized cellulose aerogels for boosting energy efficiency of glazing in buildings. *Nature Energy* , 1-16.	https://www.nature.com/articles/s41560-023-01226-7
		0.009	26.9					
		0.015	24.5					
		0.033	17.8					
		0.038	15.5					
		0.020	23.8					
		0.024	22.0					
		0.035	18.4					
**23**	Silica	0.091	10.0	SCD	Home-built hotwire	Transient	Zuo, Y. (2010). *Preparation of silica aerogels with improved mechanical properties and extremely low thermal conductivities through modified sol-gel process* (Doctoral dissertation, Massachusetts Institute of Technology).	https://dspace.mit.edu/handle/1721.1/64600
		0.108	10.3					

		0.122	10.6					
		0.123	10.7					
		0.155	11.1					
		0.140	10.9					
		0.121	10.0					
		0.099	9.8					
		0.095	9.8					
		0.091	10.0					
		0.131	9.3					
		0.106	9.3					
		0.091	10.0					
		0.087	10.1					
		0.146	11.1					
		0.128	9.1					
		0.131	9.3					
**24**	Chitosan	0.094	24.1	SCD	Home-built guarded hotplate	Steady-state	Guerrero-Alburquerque, N.,et al. (2020). Strong, machinable, and insulating chitosan–urea aerogels: toward ambient pressure drying of biopolymer aerogel monoliths. *ACS applied materials & interfaces*, *12* (19), 22037-22049.	https://pubs.acs.org/doi/full/10.1021/acsami.0c03047
		0.121	25.7	SCD				
		0.145	27.8	SCD				
		0.144	27.9	SCD				
		0.152	26.2	SCD				
		0.305	37.5	APD				
		0.361	62.5	APD				
		0.423	68.1	APD				
		0.278	41.6	APD				
		0.225	36.3	APD				
		0.292	44.5	APD				
		0.304	55.6	APD				
		0.180	37.0	APD				
		0.167	30.9	APD				
**25**	Cellulose	0.014	34.5	SCD	Home-built guarded hotplate	Steady-state	Sivaraman, D.et al. (2022). Superinsulating nanocellulose aerogels: Effect of density and nanofiber alignment. *Carbohydrate Polymers*, *292*, 119675.	https://www.sciencedirect.com/science/article/pii/S014486172200580X
		0.017	30.2					
		0.025	26.5					
		0.030	23.5					
		0.046	21.5					
		0.068	18.7					
		0.085	19.8					
		0.104	20.9					
		0.006	42.5					
		0.007	37.5					
		0.009	35.5					
		0.014	34.5					
		0.017	32.7					
		0.021	31.8					
		0.023	31.0					
		0.025	29.9					
		0.030	28.8					
		0.033	28.3					
**26**	Cellulose/PAA	0.200	70.0	FD	Hot Disk TPS 2500	Transient	Zhou, J., & Hsieh, Y. L. (2020). Nanocellulose aerogel-based porous coaxial fibers for thermal insulation. *Nano Energy*, *68*, 104305.	https://www.sciencedirect.com/science/article/pii/S2211285519310122
**27**	Cellulose/Zirconia	0.009	18.0	FD	Hot Disk TPS 2500	Transient	Wang, D. et al. (2020). Biomimetic structural cellulose nanofiber aerogels with exceptional mechanical, flame-retardant and thermal-insulating properties. *Chemical Engineering Journal*, *389*, 124449.	https://www.sciencedirect.com/science/article/pii/S138589472030440X
**28**	Alginate/Clay	0.036	33.2	FD	Hot Disk TPS 2500	Transient	Jin, H.et al. (2020). Ultralight and hydrophobic palygorskite-based aerogels with prominent thermal insulation and flame retardancy. *ACS applied materials & interfaces*, *12* (10), 11815-11824.	https://pubs.acs.org/doi/full/10.1021/acsami.9b20923
	Alginate/Clay	0.052	37.0					
**29**	Glucomannan	0.030	21.0	FD	Hot Disk Xiangyi, DER-III	Transient	Zhu, J.et al. (2019). Ultralight, hydrophobic, monolithic konjac glucomannan-silica composite aerogel with thermal insulation and mechanical properties. *Carbohydrate polymers*, *207*, 246-255.	https://www.sciencedirect.com/science/article/pii/S0144861718314048
	Glucomannan/silica	0.084	33.0	FD				
**30**	CNF	0.005	43.0	FD	Hot Disk TPS 2500	Transient	Zhou, S. et al. (2020). Elastic aerogels of cellulose nanofibers@ metal–organic frameworks for thermal insulation and fire retardancy. *Nano-Micro Letters*, *12*, 1-13.	https://link.springer.com/article/10.1007/s40820-019-0343-4
	CNF/MOF	0.003	41.0	FD				
**31**	Cellulose	0.056	36.0	SCD	Hot Disk TPS 2500	Transient	Zhang, S.et al. (2020). Thermal conductivities of cellulose diacetate based aerogels. *Cellulose*, *27*, 4555-4564.	https://link.springer.com/article/10.1007/s10570-020-03084-y
	Cellulose	0.082	34.6	SCD				
	Cellulose	0.092	31.3	SCD				
	Cellulose	0.098	33.5	SCD				
	Cellulose	0.147	36.7	SCD				
	Cellulose	0.199	43.2	SCD				
	Cellulose	0.235	50.7	SCD				
**32**	Cellulose	0.012	26.1	FD	Hot Disk TPS 2500	Transient	Gupta, P. et al. (2019). Flame retardant and thermally insulating clay based aerogel facilitated by cellulose nanofibers. *The Journal of Supercritical Fluids*, *152*, 104537.	https://www.sciencedirect.com/science/article/pii/S0896844618308751
	Cellulose/clay	0.024	36.0	FD				
**33**	Cellulose	0.055	28.0	FD	Homebuilt Laser	Transient	Song, J.,et al. (2018). Highly compressible, anisotropic aerogel with aligned cellulose nanofibers. *ACS nano*, *12* (1), 140-147.	https://pubs.acs.org/doi/full/10.1021/acsnano.7b04246
**34**	Alginate	0.028	22.0	SCD	Hotwire	Transient	Fricke, M.,et al. (2018). *U.S. Patent No. 10,017,621*. Washington, DC: U.S. Patent and Trademark Office.	https://patents.google.com/patent/US10017621B2/en
	Alginate/Starch	0.066	21.6	SCD				
	Alginate/Starch	0.058	20.4	SCD				
	Alginate/Lignin	0.062	19.4	SCD				
**35**	Pectin	0.110	22.0	SCD	DSC based	Undefined	Nešić, A et al. (2018). Pectin-based nanocomposite aerogels for potential insulated food packaging application. *Carbohydrate polymers*, *195*, 128-135.	https://www.sciencedirect.com/science/article/pii/S0144861718304703
	Pectin/TiO2	0.140	24.0	SCD				
	Pectin/TiO2	0.220	25.0	SCD				
	Pectin/TiO2	0.240	25.0	SCD				
**36**	Pectin	0.049	19.2	SCD	Customized Fox 150	Steady-state	Groult, S., & Budtova, T. (2018). Thermal conductivity/structure correlations in thermal super-insulating pectin aerogels. *Carbohydrate polymers*, *196*, 73-81.	https://www.sciencedirect.com/science/article/pii/S0144861718305484
	Pectin	0.055	20.2	SCD				
	Pectin	0.069	18.9	SCD				
	Pectin	0.069	18.6	SCD				
	Pectin	0.051	19.7	SCD				
	Pectin	0.048	20.3	SCD				
	Pectin	0.051	24.7	SCD				
	Pectin	0.053	24.6	SCD				
	Pectin	0.075	16.9	SCD				
	Pectin	0.048	20.0	SCD				
	Pectin	0.092	15.7	SCD				
	Pectin	0.127	17.1	SCD				
	Pectin	0.119	17.0	SCD				
	Pectin	0.166	20.0	SCD				

	Pectin	0.031	21.5	SCD				
	Pectin	0.048	20.0	SCD				
	Pectin	0.055	20.4	SCD				
	Pectin	0.082	21.3	SCD				
	Pectin	0.103	14.7	SCD				
	Pectin	0.143	17.7	SCD				
	Pectin	0.127	16.2	SCD				
	Pectin	0.182	21.7	SCD				
	Pectin	0.127	16.6	SCD				
	Pectin	0.089	15.7	SCD				
	Pectin	0.062	19.9	SCD				
	Pectin	0.054	21.0	SCD				
**37**	Cellulose	0.068	30.3	SCD	Netzsch LFA 467 HyperFlash	Transient	Plappert, S. F.et al. (2017). Strain hardening and pore size harmonization by uniaxial densification. *Chemistry of Materials*, *29* (16), 6630-6641.	https://pubs.acs.org/doi/full/10.1021/acs.chemmater.7b00787
	Cellulose	0.076	26.6	SCD				
	Cellulose	0.087	17.7	SCD				
	Cellulose	0.114	24.4	SCD				
**38**	Cellulose	0.002	47.7	FD	Hot Disk TPS 2500	Transient	Fan, B., et al. (2017). Fabrication of cellulose nanofiber/AlOOH aerogel for flame retardant and thermal insulation. *Materials*, *10* (3), 311.	https://www.mdpi.com/1996-1944/10/3/311
	Cellulose/AlOOH	0.003	38.5	FD				
**39**	Chitosan	0.042	22.0	SCD	EKO Instruments, HC-074	Steady-state	Takeshita, S., & Yoda, S. (2015). Chitosan aerogels: transparent, flexible thermal insulators. *Chemistry of Materials*, *27* (22), 7569-7572.	https://pubs.acs.org/doi/full/10.1021/acs.chemmater.5b03610
**40**	Chitosan	0.103	16.9	SCD	EKO Instruments, HC-074	Steady-state	Takeshita, S., & Yoda, S. (2018). Upscaled preparation of trimethylsilylated chitosan aerogel. *Industrial & Engineering Chemistry Research*, *57* (31), 10421-10430.	https://pubs.acs.org/doi/full/10.1021/acs.iecr.8b02332
		0.088	16.3					
		0.095	16.3					
		0.093	15.8					
**41**	Silica/Polyimide	0.129	23.4	SCD	Home-built guarded hotplate	Steady-state	Kantor, Z., et al. (2022). Heterogeneous silica-polyimide aerogel-in-aerogel nanocomposites. *Chemical Engineering Journal*, *443*, 136401.	https://www.sciencedirect.com/science/article/pii/S1385894722018964
		0.146	19.9					
		0.157	18.9					
		0.162	18.7					
**42**	Polyimide	0.081	30.1	SCD	Hot Disk TPS 2500	Transient	Feng, J et al. "Study on thermal conductivities of aromatic polyimide aerogels." *ACS applied materials & interfaces* 8.20 (2016): 12992-12996.	https://pubs.acs.org/doi/10.1021/acsami.6b02183
		0.114	30.9					
		0.141	31.0					
**43**	Polyimide	0.137	33.4	SCD	Hot Disk TPS 2500	Transient	Wu, Shuai, et al. "Effects of monomer rigidity on the microstructures and properties of polyimide aerogels cross-linked with low cost aminosilane." *RSC advances* 6.27 (2016): 22868-22877.	https://pubs.rsc.org/en/content/articlelanding/2016/ra/c5ra28152k
		0.124	33.6					
		0.172	49.2					
		0.246	66.8					
		0.300	45.3					
		0.284	42.2					
		0.245	38.3					
		0.246	66.8					
**44**	Polyimide	0.122	32.2	SCD	Hot Disk TPS 2500	Transient	Wu, Shuai, et al. "Solution-processable polyimide aerogels with high hydrophobicity." *Materials Letters* 176 (2016): 118-121.	https://www.sciencedirect.com/science/article/pii/S0167577X16305894
		0.137	33.4					
		0.124	33.6					
		0.172	49.5					
		0.246	59.4					
**45**	Polyimide	0.090	23.0	SCD	Undefined/Heat flow meter	Steady state	Kim, M., et al. (2018). Low shrinkage, mechanically strong polyimide hybrid aerogels containing hollow mesoporous silica nanospheres. *Composites Science and Technology*, *165*, 355-361.	https://www.sciencedirect.com/science/article/pii/S026635381830931X
**46**	Polyimide/Organosilica	0.380	43.0	SCD	Hot Disk TPS 2500	Transient	Zhang, Z.,et al. (2020). Effect of different chemical liquid deposition methods on the microstructure and properties of polyimide-polyvinylpolymethylsiloxane composite aerogels. *The Journal of Supercritical Fluids*, *160*, 104811.	https://www.sciencedirect.com/science/article/pii/S0896844620300620
		0.420	47.0					
		0.360	55.0					
		0.640	67.0					
		0.660	71.0					
		0.470	78.0					
**47**	Polyimide/silica	0.080	22.0	APD	Hot Disk TPS 2500	Transient	Wei Fan, et al. Lightweight, strong, and super-thermal insulating polyimide composite	https://www.sciencedirect.com/science/article/pii/S0266353818323479
**48**	Polyimide/halloysite	0.065	42.0	APD	Hot Disk Xiangyi, DER-III	Transient	Zhao, F.et al. (2021). Preparation of functionalized halloysite reinforced polyimide composite aerogels with excellent thermal insulation properties. *Applied Clay Science*, *211*, 106200.	https://www.sciencedirect.com/science/article/pii/S0169131721002246
		0.067	38.0					
		0.072	40.0					
**49**	Polyimide/Aramid fibers	0.014	32.0	FD	LFA 467 Nano-Flash	Transient	Xu, G., et al. (2020). Highly compressible and anisotropic polyimide aerogels containing aramid nanofibers. *Reactive and Functional Polymers*, *154*, 104672.	https://www.sciencedirect.com/science/article/pii/S138151482030482X
		0.014	22.0					
		0.013	25.0					
		0.012	27.0					
**50**	Polyimide/hydroxyapatite	0.045	34.0	FD	Hot Disk Xiangyi, DER-III	Transient	Zhu, J., et al. (2021). Highly elastic and robust hydroxyapatite nanowires/polyimide composite aerogel with anisotropic structure for thermal insulation. Composites Part B: Engineering, 223, 109081.	https://www.sciencedirect.com/science/article/pii/S1359836821004650
		0.036	33.0					
		0.032	31.0					
		0.041	37.0					
**51**	Polyimide/glass fibers	0.151	25.0	SCD	Netzsch, HFM436 Lambda	Steady state	Zhu, Z. et al. (2019). Fiber reinforced polyimide aerogel composites with high mechanical strength for high temperature insulation. *Macromolecular Materials and Engineering*, *304* (5), 1800676.	https://onlinelibrary.wiley.com/doi/full/10.1002/mame.201800676
		0.164	27.0					
		0.177	29.0					
**52**	Polyimide/Silica/Glass	0.120	26.5	SCD	TC3000, XIATECH Hotwire	Transient	Fei, Z. et al. (2018). Preparation and characterization of glass fiber/polyimide/SiO 2 composite aerogels with high specific surface area. *Journal of Materials Science*, *53*, 12885-12893.	https://link.springer.com/article/10.1007/s10853-018-2553-4
		0.124	27.2					
		0.116	26.3					
		0.132	27.5					
		0.145	28.2					
**53**	Polyimide/Silica	0.168	37.1	SCD	Hot Disk TPS 2500	Transient	Wu, S.et al. (2016). Silica-aerogel-powders "jammed" polyimide aerogels with excellent hydrophobicity and conversion to ultra-light polyimide aerogel. *RSC advances*, *6* (63), 58268-58278.	https://pubs.rsc.org/en/content/articlehtml/2016/ra/c6ra11801a
		0.152	30.7					
		0.119	28.9					
		0.092	28.3					
		0.023	37.1					
		0.156	27.2					
		0.136	32.1					
**54**	Polyimide/Ceramics	0.249	45.0	SCD	Hot Disk Xiangyi, DER-III	Transient	Hou, X.et al. (2021). SiC whiskers-reinforced polyimide aerogel composites with robust compressive properties and efficient thermal insulation performance. *Journal of Applied Polymer Science*, *138* (8), 49892.	https://onlinelibrary.wiley.com/doi/full/10.1002/app.49892
		0.280	49.7					
		0.238	36.3					
		0.211	35.0					
**55**	Polyimide/Organosilica	0.025	30.0	FD	Hot Disk TPS 2500	Transient	Tian, J.,et al. (2022). Highly flexible and compressible polyimide/silica aerogels with integrated double network for thermal insulation and fire-retardancy. *Journal of Materials Science & Technology*, *105*, 194-202.	https://www.sciencedirect.com/science/article/pii/S1005030221007581
		0.040	25.0					
		0.050	23.0					
		0.130	20.0					
**56**	Melamine-Formaldehyde/fibers	0.158	24.0	SCD	Netzsch, HFM436 Lambda	Steady state	Hayase, G. (2023) Boehmite Nanofiber–Melamine–Formaldehyde Composite Aerogels and Derivatives for Thermal Insulation and Optical Applications. ACS Applied Nanomaterials. https://doi.org/10.1021/acsanm.3c01980	https://pubs.acs.org/doi/10.1021/acsanm.3c01979
		0.139	21.4					

		0.136	26.7					
**57**	Organosilica	0.450	56.0	SCD	Netzsch, HFM436 Lambda	Steady state	Hayase, G. et al. (2014). The thermal conductivity of polymethylsilsesquioxane aerogels and xerogels with varied pore sizes for practical application as thermal superinsulators. *J. Mat. Chem. A*, *2* (18), 6525-6531.	https://pubs.rsc.org/en/content/articlehtml/2014/ta/c3ta15094a
		0.450	52.0					
		0.410	36.0					
		0.360	34.0					
		0.290	21.0					
		0.270	18.0					
**58**	Organosilica/cellulose	0.020	24.3	SCD	Netzsch, HFM436 Lambda	Steady state	Hayase, G.et al. (2014). Polymethylsilsesquioxane–cellulose nanofiber biocomposite aerogels with high thermal insulation, bendability, and superhydrophobicity. *ACS applied materials & interfaces*, *6* (12), 9466-9471.	https://pubs.acs.org/doi/full/10.1021/am501822y
		0.039	21.7					
		0.097	18.8					
		0.142	15.3					
		0.186	16.2					
		0.040	22.5					
		0.135	14.9					
**59**	Organosilica	0.160	15.3	APD	Netzsch, HFM436 Lambda	Steady state	Shimizu, T., et al. (2016). Transparent, highly insulating polyethyl-and polyvinylsilsesquioxane aerogels: mechanical improvements by vulcanization for ambient pressure drying. *Chemistry of Materials*, *28* (19), 6860-6868.	https://pubs.acs.org/doi/pdf/10.1021/acs.chemmater.6b01936
**60**	Clay/PVA	0.082	41.0	FD	Netzsch, HFM436 Lambda	Steady state	Skaropoulou, A.et al. (2022). The effect of synthesis parameters on density and thermal conductivity of MMT based aerogel. Application of experimental design model. *Materials Today: Proceedings*, *58*, 1005-1010.	https://www.sciencedirect.com/science/article/pii/S2214785321082365
		0.064	39.0					
		0.048	38.0					
**61**	rGO	0.009	19.0	FD	Netzsch, HFM436 Lambda	Steady state	Zu, G.et al. (2019). Superelastic multifunctional aminosilane-crosslinked graphene aerogels for high thermal insulation, three-component separation, and strain/pressure-sensing arrays. *ACS AMI*, *11* (46), 43533-43542.	https://pubs.acs.org/doi/pdf/10.1021/acsami.9b16746
		0.003	19.0					
**62**	rGO	0.005	21.0	FD	Hot Disk TPS 2500	Transient	Yuan, Y.,et al. Lightweight, thermally insulating and stiff carbon honeycomb-induced graphene composite foams with a horizontal laminated structure for electromagnetic interference shielding. *Carbon*, *123*, 223-232.	https://www.sciencedirect.com/science/article/pii/S000862231730742X
		0.096	23.0					
		0.019	28.0					
**63**	Polyethylene	0.012	53.0	SCD	Home-built guarded hotplate	Steady-state	Leroy, A.,et al. (2022). Thermal transport in solar-reflecting and infrared-transparent polyethylene aerogels. *International Journal of Heat and Mass Transfer*, *184*, 122307.	https://www.sciencedirect.com/science/article/pii/S001793102101406X
		0.016	52.5					
		0.024	57.0					
		0.053	54.0					
		0.081	57.0					
		0.015	28.0					
		0.182	28.5					
		0.027	32.0					
		0.054	34.0					
		0.082	41.0					
**64**	Silica	0.082	16.3	SCD	Home-built	Steady state	Lee, D.,et al. (1995). Thermal characterization of carbon-opacified silica aerogels. *Journal of Non-Crystalline Solids*, *186*, 285-290.	https://www.sciencedirect.com/science/article/pii/0022309395000550
		0.077	14.6					
		0.086	14.6					
		0.098	14.4					
		0.090	13.6					
**65**	Resorcinol-Formaldehyde	0.060	20.2	SCD	Home-built hotwire	Transient	Lu, X., et al. (1995). Correlation between structure and thermal conductivity of organic aerogels. *Journal of Non-Crystalline Solids*, *188* (3), 226-234.	https://www.sciencedirect.com/science/article/pii/0022309395001913
		0.074	19.6					
		0.074	15.6					
		0.082	16.0					
		0.102	16.7					
		0.120	14.4					
		0.128	13.8					
		0.138	15.0					
		0.158	11.9					
		0.180	15.0					
		0.184	13.3					
		0.280	16.7					
		0.282	20.6					
		0.304	18.1					
**66**	Alginate/Cellulose	0.015	31.5	FD	Home-built guarded hotplate	Steady-state	Berglund, L., et al. (2021). Seaweed-derived alginate–cellulose nanofiber aerogel for insulation applications. *ACS Applied Materials & Interfaces*, *13* (29), 34899-34909.	https://pubs.acs.org/doi/full/10.1021/acsami.1c07954
**67**	Polyimide/silica	0.135	20.3	SCD	Home-built guarded hotplate	Steady-state	Wu, T.,et al. (2023). 3D Printed Polyimide Nanocomposite Aerogels for Electromagnetic Interference Shielding and Thermal Management. *Advanced Materials Technologies*, 2202155.	https://onlinelibrary.wiley.com/doi/full/10.1002/admt.202202155
**68**	Polyurethane	0.142	12.6		Customized Fox 314	Steady-state	Merillas, B., et al. (2022). Super-Insulating Transparent Polyisocyanurate-Polyurethane Aerogels: Analysis of Thermal Conductivity and Mechanical Properties. *Nanomaterials*, *12* (14), 2409.	https://www.mdpi.com/2079-4991/12/14/2409
		0.165	11.7					
		0.154	12.6					
		0.141	13.0					
		0.131	14.3					
		0.101	19.8					
		0.102	24.6					
		0.102	24.2					
**69**	Polyurethane	0.042	52.0	FD	Heat flow meter KD2Pro	Transient	Cantero, D., et al. Synthesis of waterborne polyurethane aerogels-like materials via freeze-drying: an innovative approach. J Mater Sci (2023).	https://link.springer.com/article/10.1007/s10853-023-08579-0
		0.030	46.0					
		0.027	46.0					
		0.021	35.0					
**70**	Cellulose/MTMS	0.003	28.6	FD	Hot Disk TPS 2500	Transient	Guo. W. et al. Ultra-light-weight, anti-flammable and water-proof cellulosicaerogels for thermal insulation applications. International Journal of Biological Macromolecules. 10.1016/j.ijbiomac.2023.125343	https://www.sciencedirect.com/science/article/pii/S0141813023022377
		0.004	28.4					
		0.003	33.2					
		0.004	35.7					
		0.004	39.7					
		0.005	40.3					
**71**	Cellulose	0.069	16.0	SCD	LFA 467 Netsch	Transient	Zhao P.et al. Flexible and Transparent Bagasse Aerogels for Thermal RegulationGlazing. ACS Sustainable Chemistry & Engineering. 10/1021/acssuschemeng.3c01600	https://pubs.acs.org/doi/pdf/10.1021/acssuschemeng.3c01600
		0.074	19.0					
		0.067	17.0					
**72**	Polyimide/Silica/Fibers	0.101	21.7	SCD	Home-built guarded hotplate	Steady-state	Häusermann D. et al. Firedrone: Multi-environment thermally agnostic aerial robot. Advanced Intelligent Systems 10.1002/aisy.202300101	https://onlinelibrary.wiley.com/doi/full/10.1002/aisy.202300101
		0.0902	22.7					
		0.089	21.4					
		0.069	25.3					
		0.068	25.6					
		0.093	28.8					
		0.065	26.2					
		0.1677	20.1					
		0.1677	20.1					
		0.1623	18.1					
		0.1634	17					

		0.114	16.9					
		0.102	19.9					
		0.144	17.9					
		0.151	20.9					
		0.158	23.8					
		0.16	22.9					
		0.16	22.4					
		0.16	23.3					
		0.18	24.6					
		0.16	23.4					
		0.17	25.3					
		0.175	21.3					
		0.18	29.7					
		0.138	21.7					
**73**	Polyurethane	0.108	19.0	SCD	Taurus HFM	Steady state	Ebert, H. P.et al. (2021). Intercomparison of thermal conductivity measurements on a nanoporous organic aerogel. *International Journal of Thermophysics* , *42* , 1-18.	https://link.springer.com/article/10.1007/s10765-020-02775-9
		0.108	18.8	SCD	Home-built guarded hotplate	Steady state		
		0.108	19.3	SCD	TA Instruments HFM	Steady state		
		0.108	19.2	SCD	Undefined/Guarded hotplate	Steady state		
		0.108	19.5	SCD	home-build guarded hotplate	Steady state		
		0.108	19.6	SCD	home-build guarded hotplate	Steady state		
		0.108	19.3	SCD	home-build guarded hotplate	Steady state		
		0.108	19.6	SCD	home-build guarded hotplate	Steady state		
		0.108	19.6	SCD	home-build guarded hotplate	Steady state		
		0.108	19.5	SCD	Netzsch, HFM 436 Lambda	Steady state		
		0.108	33.1	SCD	Hot Disk TPS 2500	Transient		
**74**	Silica/fibers	0.094	18.0	APD	Netzsch HFM 436/3/1E	Steady state	Martinez, R. G., et al. (2016). Thermal assessment of ambient pressure dried silica aerogel composite boards at laboratory and field scale. *Energy and Buildings* , *128* , 111-118.	https://www.sciencedirect.com/science/article/pii/S0378778816305618
		0.100	16.0		Netzsch HFM 436/3/1E	Steady state		
		0.114	16.0		Home-built guarded hotplate	Steady-state		
		0.100	13.9		Home-built guarded hotplate	Steady-state		
		0.102	14.5		Home-built guarded hotplate	Steady-state		
		0.103	14.2		Home-built guarded hotplate	Steady-state		
**75**	Silica	0.064	18.1	SCD	Home-built guarded hotplate	Steady-state	Huber, L., et al. (2017). Fast and Minimal-Solvent Production of Superinsulating Silica Aerogel Granulate. *Angewandte Chemie International Edition* , *56* (17), 4753-4756.	https://onlinelibrary.wiley.com/doi/full/10.1002/anie.201700836
		0.113	15.6					
		0.134	14.8					
		0.154	17.3					
		0.213	17.4					
**76**	Polyurea	0.040	27.1	SCD	Hot-wire	Transient	Weigold, L., et al. (2013). Correlation of microstructure and thermal conductivity in nanoporous solids: the case of polyurea aerogels synthesized from an aliphatic tri-isocyanate and water. *JNCS* , *368* , 105-111.	https://www.sciencedirect.com/science/article/pii/S0022309313001294
		0.064	25.0					
		0.121	25.6					
		0.177	32.0					
		0.298	38.1					
		0.530	66.2					
**77**	Silica/fibers	0.131	19.5	SCD	Heat flow meter	Steady-state	Lakatos, Á.,et al. (2021). Experimental verification of thermal properties of the aerogel blanket. *Case Studies in Thermal Engineering* , *25* , 100966.	https://www.sciencedirect.com/science/article/pii/S2214157X21001295
**78**	Cellulose	0.005	26.6	SCD	Home-built guarded hotplate	Steady-state	Obori, M. et al. (2019). Parametric model to analyze the components of the thermal conductivity of a cellulose-nanofibril aerogel. *Physical Review Applied* , *11* (2), 024044.	https://journals.aps.org/prapplied/pdf/10.1103/PhysRevApplied.11.024044
		0.009	25.8					
		0.014	24.4					
		0.019	24.0					
		0.022	20.7					
**79**	Silica/fibers	0.130	15.5	SCD	HFM436 Netzsch	Steady-state	Hoseini, A.et al. (2016). Aerogel blankets: From mathematical modeling to material characterization and experimental analysis. *International Journal of Heat and Mass Transfer* , *93* , 1124-1131.	https://www.sciencedirect.com/science/article/pii/S0017931015301253
		0.070	21.7	APD				
**80**	Silica/fibers	0.365	30.0	SCD	Hot Disk TPS 2500	Transient	Zhang, H., et al. (2017). Thermal conductivity of fiber and opacifier loaded silica aerogel composite. *International Journal of Heat and Mass Transfer* , *115* , 21-31.	https://www.sciencedirect.com/science/article/pii/S0017931017321877
		0.354	33.0					
		0.368	33.5					
		0.387	34.0					
		0.414	35.0					
		0.391	33.0					
		0.386	33.5					
		0.388	34.0					
		0.487	32.0					
		0.429	35.0					
**81**	Silica/fibers	0.162	22.1	APD	Hot Disk TPS 2500	Transient	Li, Z.,et al. (2016). Aramid fibers reinforced silica aerogel composites with low thermal conductivity and improved mechanical performance. *Composites Part A: Applied Science and Manufacturing* , *84* , 316-325.	https://www.sciencedirect.com/science/article/pii/S1359835X16000774
		0.152	22.3					
		0.144	22.1					
		0.145	23.2					
		0.140	23.5					
**82**	Zirconia/silica/ceramic	0.330	101.5	SCD	Hotwire	Transient	He, J., et al. (2016). Ultra-low thermal conductivity and high strength of aerogels/fibrous ceramic composites. *Journal of the European Ceramic Society* , *36* (6), 1487-1493.	https://www.sciencedirect.com/science/article/pii/S0955221915302302
		0.450	52.0					
**83**	Alumina/silica	0.150	27.0	SCD	DER-III Hot Disk	Transient	Hou, X., et al. (2017). Novel whisker-reinforced Al2O3–SiO2 aerogel composites with ultra-low thermal conductivity. *Ceramics International* , *43* (12), 9547-9551.	https://www.sciencedirect.com/science/article/pii/S0272884217306454
		0.230	35.0					
		0.350	40.0					
		0.410	49.0					
		0.520	64.0					
		0.450	52.0					
**84**	GO/SiC	0.014	25.0	FD	HCDR-S hot-wire	Transient	Song, L., et al. (2022). Low weight, low thermal conductivity, and highly efficient electromagnetic wave absorption of three-dimensional graphene/SiC-nanosheets aerogel. *Composites Part A* , *158* , 106980.	https://www.sciencedirect.com/science/article/pii/S1359835X22001695
		0.022	27.0					
		0.024	29.0					
		0.025	30.0					
		0.026	33.0					
		0.028	35.0					
**85**	Cellulose/starch	0.020	51.0	FD	Hot Disk TPS 2500	Transient	Wang, Y.,et al. (2018). Thermal conductivity, structure and mechanical properties of konjac glucomannan/starch based aerogel strengthened by wheat straw. *Carbohydrate polymers* , *197* , 284-291.	https://www.sciencedirect.com/science/article/pii/S0144861718306568
		0.041	49.0					
		0.052	53.0					
		0.041	47.0					
		0.051	52.0					
		0.036	51.0					
		0.047	52.0					
		0.039	49.0					

		0.044	53.0					
		0.043	46.0					
**86**	Silica/organosilica	0.129	48.0	APD	Hot Disk TPS 2500	Transient	Zhang, Y.et al. (2021). Rapid synthesis of dual-mesoporous silica aerogel with excellent adsorption capacity and ultra-low thermal conductivity. *Journal of Non-Crystalline Solids* , *555* , 120547.	https://www.sciencedirect.com/science/article/pii/S0022309320306578
		0.126	40.0					
		0.112	22.0					
		0.632	49.0					
		1.164	92.0					
**87**	Silica/zirconia	0.160	23.5	SCD	DER-III Hot Disk	Transient	Hou, X., et al. (2018). An ultralight silica-modified ZrO2–SiO2 aerogel composite with ultra-low thermal conductivity and enhanced mechanical strength. *Scripta Materialia* , *143* , 113-116.	https://www.sciencedirect.com/science/article/pii/S1359646217305407
		0.230	23.6					
		0.290	26.2					
		0.330	29.6					
